# Healthcare needs in elderly patients with chronic heart failure in view of a personalized blended collaborative care intervention: a cross sectional study

**DOI:** 10.3389/fcvm.2024.1332356

**Published:** 2024-03-13

**Authors:** Sara Gostoli, Francesco Bernardini, Regina Subach, Petra Engelmann, Tiny Jaarsma, Frida Andréasson, Sanne Rasmussen, Trine Thilsing, Natasja Eilerskov, Barbara Bordoni, Diego Della Riva, Stefano Urbinati, Sebastian Kohlmann, Chiara Rafanelli

**Affiliations:** ^1^ Department of Psychology “Renzo Canestrari”, University of Bologna, Bologna, Italy; ^2^ Department of Psychosomatic Medicine, University Medical Center Hamburg-Eppendorf, Hamburg, Germany; ^3^ Department of Health, Medicine and Caring Sciences (HMV), Linköping University, Linköping, Sweden; ^4^ Research Unit of General Practice, Institute of Public Health, University of Southern Denmark, Odense, Denmark; ^5^ Division of Cardiology, Bellaria Hospital, AUSL Bologna, Bologna, Italy; ^6^ Department of Psychology, University of Southern Denmark, Odense, Denmark

**Keywords:** heart failure, patient preferences, multimorbidity, aged, patient-centered care, care manager, blended collaborative care, healthcare needs

## Abstract

**Introduction:**

Few studies explored healthcare needs of elderly heart failure (HF) patients with comorbidities in view of a personalized intervention conducted by Care Managers (CM) in the framework of Blended Collaborative Care (BCC). The aims of the present study were to: (1) identify perceived healthcare needs/preferences in elderly patients with HF prior to a CM intervention; (2) investigate possible associations between healthcare needs/preferences, sociodemographic variables (age; sex) and number of comorbidities.

**Method:**

Patients aged 65 years or more affected by HF with at least 2 medical comorbidities were enrolled in the study. They were assessed by structured interviewing with colored cue cards that represented six main topics including education, individual tailoring of treatment, monitoring, support, coordination, and communication, related to healthcare needs and preferences.

**Results:**

Thirty-three patients (Italy = 21, Denmark = 7, Germany = 5; mean age = 75.2 ± 7.7 years; males 63.6%) were enrolled from June 2021 to February 2022. Major identified needs included: HF information (education), patients' involvement in treatment-related management (individual tailoring of treatment), regular checks of HF symptoms (monitoring), general practitioner update by a CM about progression of symptoms and health behaviors (coordination), and telephone contacts with the CM (communication). Regarding communication modalities with a CM, males preferred phone calls (*χ*^2 ^= 6.291, *p *= 0.043) and mobile messaging services (*χ*^2 ^= 9.647, *p *= 0.008), whereas females preferred in-person meetings and a patient dashboard. No differences in needs and preferences according to age and number of comorbidities were found.

**Discussion:**

The findings highlight specific healthcare needs and preferences in older HF multimorbid patients, allowing a more personalized intervention delivered by CM in the framework of BCC.

## 1 Introduction

The 2019 Heart Failure Association (HFA) ATLAS provided insights about heart failure (HF) in Europe. It found that, on average, there were 3.2 cases per 1,000 person-years, with a range from ≤2 cases in Italy and Denmark to >6 cases in Germany. The prevalence of HF cases increases with age, with rates at 0.6 per 1,000 for those under 65 years of age, escalating to 28 per 1,000 among those aged 65 and above (1). This means that HF is becoming a bigger challenge for modern healthcare, especially as the population ages (2).

Elderly patients with HF represent a vulnerable group with a wide range of somatic and mental comorbidities (3, 4) leading to low health related quality of life (5). This complicated clinical picture may result in complex drug-to-drug interactions due to polypharmacy (6–8), poor medical adherence (6) and increased hospitalizations and mortality rates (8, 9). All of these factors contribute to a higher dependency in self-care and daily activities (10). Consequently, there is a need to comprehensively assist and treat patients with HF through the implementation of person-centered and integrated care approach, such as Blended Collaborative Care (BCC) (11–13).

BCC is a promising patient-centered healthcare model that integrates collaborative care strategy with the involvement of a Care Manager (CM), in treatment process of chronic disorders. It has shown beneficial effects on various conditions, such as diabetes (14), chronic obstructive pulmonary disease (15), cancer (16), HF (17), and mental health treatment programs. Moreover, it aimed to improve chronic illness care for patients with multiple comorbidities, including psychological distress (18).

BCC is based on Wagner's Chronic Care Model (19) but it also considers patient's multimorbidity in addition to chronic care. It involves nurses as CMs who regularly and proactively follow-up patients in order to educate them about their illness and adherence to treatment options, monitor critical symptoms, support them in integrating health behavior in their daily lives, offer brief interventions for psychological distress, communicate across all providers, and connect to community resources. Shared-decision making and motivational interventions, as communication techniques, are used by the CMs, who work closely with patient's general practitioner (GP) and are supervised by a specialist team that monitors evidence-based treatment plans and possible gaps.

To provide such individualized support, several authors highlighted the importance of taking into consideration HF patients' needs and preferences from their and/or their carers' perspectives (20–22). By tailoring healthcare to patients' specific needs, BCC provides personalized support, better coordination and has potential to improve overall well-being and quality of life (11–13). According to review of the literature, there is a lack of studies investigating chronically ill patients' needs and preferences in view of BCC, which is also the case for elderly HF patients. The existing literature regarding needs of elderly patients with HF is based both on qualitative investigations, which provide an explorative view on them, and quantitative studies based on validated questionnaires (20, 21). Therefore, investigating elderly HF patients’ perspective on their healthcare needs is crucial for delivering personalized care they require. To gather information regarding HF patients' educational needs, an assessment methodology based on cards was used and reported by Luniewski et al. (23). Additionally, Griber et al. (24) outlined that the use of colored cards in educational environment may support and strengthen memorization and information processing among elderly. According to the literature, the most frequent healthcare needs of geriatric HF patients with comorbidities mainly concern communication, information, social support, self-management and individualized care (20–22), which are reported to change along with disease progression.

To the best of our knowledge, few studies have provided information on healthcare needs of elderly HF patients with comorbidities in terms of how a CM could improve their health care in the framework of a BCC intervention.

Based on the above mentioned premises, the present study aimed to (1) identify perceived healthcare needs and preferences among elderly patients with HF and multiple comorbidities, to be addressed by a CM in the context of the ESCAPE BCC (25); (2) investigate possible associations between patients' needs/preferences, sociodemographic variables (age; sex) and number of comorbidities.

## 2 Materials and methods

### 2.1 Design

This study is exploratory descriptive cross-sectional hypothesis-generating research. It refers to the Patient Public Involvement phase within the framework of a large, international and multi-center trial entitled “Evaluation of a patient-centered biopsychosocial blended collaborative care pathway for the treatment of multi-morbid elderly patients” (ESCAPE project; Horizon 2020; Grant Agreement No 945377), where identified patients' needs will be incorporated into an integrated care program (BCC). The study has been registered at the University of Göttingen Medical Centre (UMG Reg. No 02853) and the German Clinical Trials Register (DRKS00025120). Specifically, the ESCAPE intervention aims to improve quality of life of HF patients having at least two other chronic somatic comorbidities and psychological distress, by means of the implementation of a combined healthcare intervention. This program involves the introduction of a nurse as a CM, whose role would be to promote communication between health care professionals, patients and carers, and to assist and support patients and carers in various aspects related to disease management (25). In the present study, patients were asked, with the support of cue cards, to share their perspective regarding their needs and preferences in relation to the management of their disease by CM in the context of BCC. The results have guided the development of the CM interventions within the ESCAPE project (25).

The present study followed the recommendations of the Declaration of Helsinki and was approved by all the local Ethics Committees. All participants were fully informed about the study, the voluntary nature of their participation, confidentiality and anonymity, and they all gave their written consent to participate.

### 2.2 Sample

A convenience sample (i.e., easily accessible and/or readily available for the study) (26) of HF patients meeting inclusion/exclusion criteria was enrolled from June 2021 to February 2022 in three European countries: Italy, Denmark, and Germany. Inclusion criteria were: (a) a diagnosis of chronic HF, clinically confirmed by a cardiologist or internist in a written medical report. In case the diagnosis of HF was not documented in any written record, the diagnostic criteria outlined in the current ESC guidelines (27) were used, sourcing information from both medical records and the patient; (b) at least two other medically diagnosed chronic comorbidities (e.g., diabetes, cancer, kidney failure) (c) age ≥65 years, (d) being able to provide written informed consent. Exclusion criteria were: (a) anticipated life expectancy less than one year due to causes other than HF (e.g., terminal stage of cancer), as established by the healthcare provider; (b) communication difficulties (e.g., speech and/or hearing problems, no means of contact, such as telephone); (c) severe mental disorders needing specific psychiatric treatment and/or interfering with the study treatment such as bipolar disorder, active suicidality, schizophrenia and dementia. Current psychosomatic or psychotherapeutic treatment was not an exclusion criterion. Inclusion and exclusion criteria were verified through patients’ medical records. The recruitment was conducted in hospitals or GP offices. The responsible centers among those participating in the ESCAPE project (25) were selected based on their availability in recruiting HF patients.

### 2.3 Assessment

Socio-demographic data and clinical characteristics were collected with a specifically designed questionnaire, which included: age, sex, educational and marital status, physical measurements such as weight, height, blood pressure and cholesterol, comorbidities and medical conditions, years with HF and smoking habits. Researchers from the ESCAPE team in each country conducted data collection independently.

**Figure 1:**
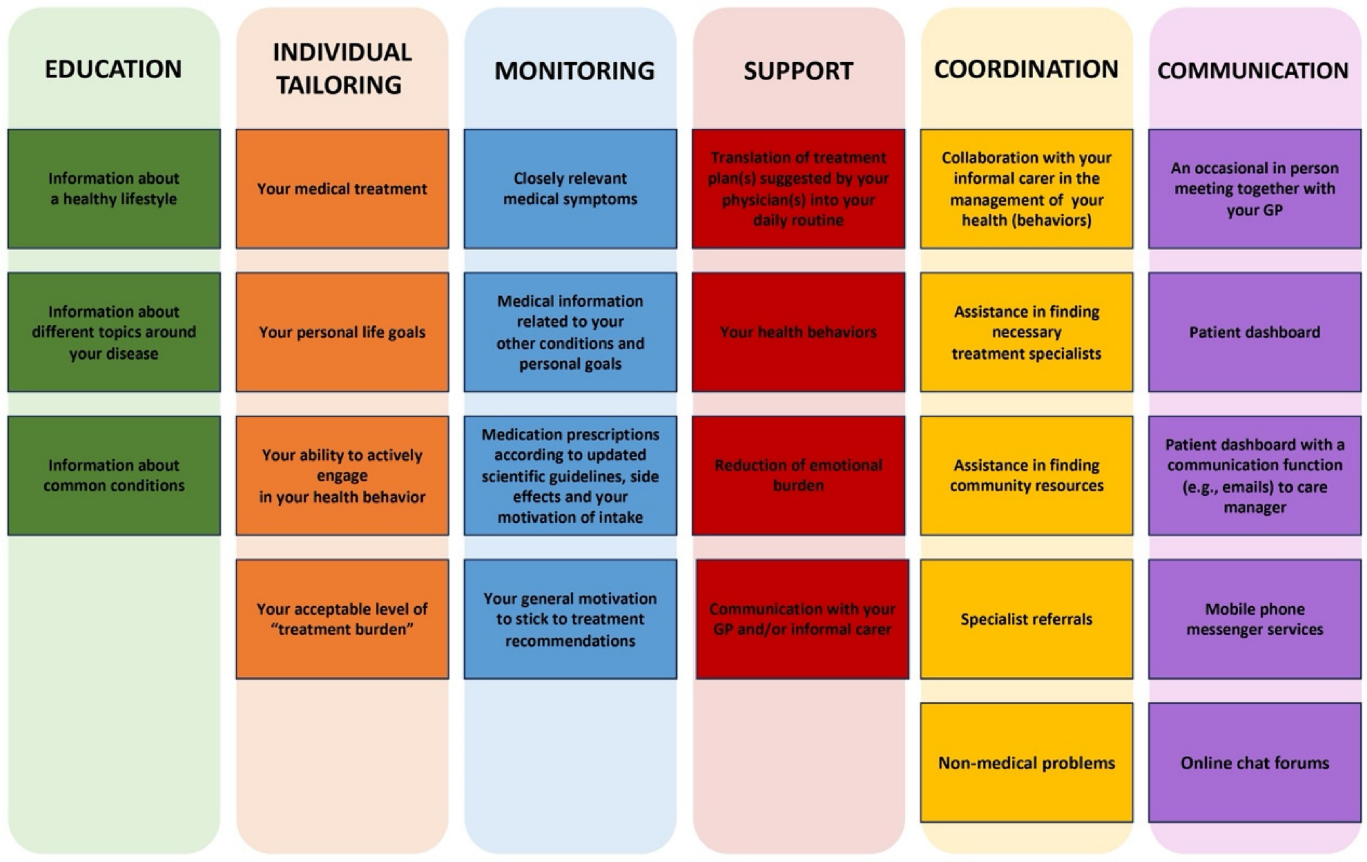
Example of colored cue cards used to collect patients’ healthcare needs and preferences to be addressed by a CM in the framework of ESCAPE BCC.

According to recommendations and experience from Herbeck Belnap and colleagues' study (12), patients' needs and preferences were clustered into six main areas, such as education, individual tailoring of treatment, monitoring, support, coordination and communication, together with additional sub-topics (see Table 1). The topics were used to guide the identification of patients' and carers' needs and the creation of individualized profiles for further better customization of a novel international ESCAPE intervention in the framework of BCC (28). In the present study, the methodology used to collect quantitative data involved cue cards (see Figure 1). Specifically, forty-five cards, divided into different colors according to the cited 6 main topics, allowed the interviewer to introduce and present the topics and sub-topics to the patients, in order to ask questions consistently and comprehensibly. All the topics presented through the cards investigated patients' needs regarding the management of their disease, which could be addressed by a CM. Patients were asked to identify their priorities among sub-topics (cards on areas of interest) in each topic, and among the options (cards on subjects related to sub-topic) within each preferred sub-topic. For example, when addressing needs related to education, patients were asked: “About which area would you like to get more information?”, consequently green cards representing “Lifestyle”, “Topics” and “Conditions” were presented to the patient. The colored cue cards were administered during assessment in person and by video calls. When assessment took place by phone, the interviewer verbally described the cards and asked questions based on the descriptions. The approximate length of the data collection appointments ranged from 35 to 90 min each. During cards administration, in case of non-response to a given topic, patients were asked to skip to the subsequent topic. The collected data were quantified by taking into account each preference within the topics and sub-topics the patient reported during the assessment.

**Table 1:** Cue cards description.

Topic	Questions & sub-topics
Education	Which area would you like to get more information about? ⚬ Generic: Information about a healthy lifestyle (e.g., exercise, diet, stress, sleep) ⚬ Targeted: Information about different topics around your disease (e.g., heart failure, emotional distress, having several medical conditions at the same time) ⚬ Specific: Information about common conditions (e.g., hypertension, diabetes type II, osteoarthritis, chronic obstructive pulmonary disease, chronic kidney disease) Which is the most important thing to get more information about, out of each card? Which means of communication do you prefer when it comes to information about your health? ⚬ Text on a website ⚬ Brochure by medical organizations ⚬ Video clip ⚬ Audio file ⚬ Education through your Care Manager
Individual tailoring of treatment	In which area do you want your treatment to be more personalized, that is more tailored to your specific living conditions? ⚬ Your medical treatment (e.g., changes in treatment, consideration of alternative treatments, more centralized treatment) ⚬ Your personal life (e.g., quality of life, social life, ability to work, leisure time activities) ⚬ Your ability to actively engage in improving your health (e.g., physical activity, change diet, engage in therapies, self-management)
Monitoring	Which aspect of your diseases do you want your Care Manager to keep an eye on? ⚬ Symptoms (e.g., shortness of breath, weight gain, blood pressure, glucose, LDL and other lab results) ⚬ Medical information related to your other conditions and personal life (e.g., stress, depression, anxiety, quality of life) ⚬ Medication prescriptions according to updated scientific guidelines and side effects ⚬ How you are following the treatment recommendations
Support	In which area do you need support by your Care Manager? ⚬ Translation of treatment plan(s) suggested by your physician(s) into your daily routine ⚬ Your health behaviours (healthy sleeping habits, diet diary, walk regimen) ⚬ Reduction of emotional burden ⚬ Communication with your GP and/or informal carer
Coordination	Which aspect should your Care Manager help coordinate? ⚬ Regular updates of the progression of your symptoms and health to your GP ⚬ Collaboration with your informal carer in the management of your health ⚬ Assistance in finding necessary treatment specialists ⚬ Assistance in finding community resources (self-help groups, cardiac groups) ⚬ Specialist referrals ⚬ Non-medical problems (transportation, payment for medication, financial stress)
Communication	How would you like to communicate with your Care Manager? ⚬ An occasional in person meeting (together with your GP) ⚬ Telephone ⚬ Video call In addition to those contacts, what other communication paths would you like to have? ⚬ Patient dashboard (a screen to track/view relevant symptoms, upcoming appointments) ⚬ Patient dashboard with a communication function (e.g., emails) to Care Manager ⚬ Mobile phone messenger services (e.g., text messages to send reminders for appointments, health behavior, medication intake) ⚬ Online chat forums (to exchange with other patients with similar health conditions)

### 2.4 Statistical analyses

Data were analyzed using SPSS 26.0 (29). In the total sample, descriptive analyses were performed. Socio-demographic and medical characteristics, needs and preferences, as assessed by cue cards, were presented as frequencies and means (±SD). Non-responses about needs and preferences were not considered for statistical purposes. Among patients' responses, only the first priority card was taken into account. Chi-square test, applied to contingency tables, was used to compare needs/preferences with sex, age classes (e.g., 65–74; ≥75) and number of medical comorbidities (e.g., 2 comorbidities; ≥3 comorbidities). Significance level was set at 0.05. Missing data were handled by complete case analysis, namely only the cases with complete data were analyzed, whereas individuals with missing data on any of the included variables were dropped from the analyses (30).

## 3 Results

The present study included 33 patients with HF. In Italy, 21 patients were recruited at a hospital division of cardiology; 16 patients were interviewed at the hospital and 5 remotely. In Denmark, 7 patients were enrolled at their GP's office and were interviewed in person at their home. In Germany, 5 patients were recruited via self-help groups, at general medicine and cardiology departments of university hospitals; 4 patients were interviewed online (WebEx) and 1 at a university hospital psychosomatic department. In Denmark and Germany, all invited patients participated in the study, whereas in Italy 6 patients refused to be enrolled due to lack of interest. The mean age was 75.2 (SD* *= 7.7) years, whereas the median age was 73 years (ranging from 65 to 91 years). Twenty-one patients (64%) were male. Of the total sample, 64% (*N *= 21) were Italian, 21% (*N *= 7) Danish and 15% (*N *= 5) German. The majority of the patients who participated in the study had NYHA Class II symptoms (76.2%) and reported cardiac arrhythmia (57.6%). Among the participants, from 12% to 24% were uncertain or did not give an answer about their comorbid medical conditions. Regarding education, the average school attendance was 8.7 (SD* *= 4.2) years (that are equivalent to 9th grade in the US). All patients were retired (100%) and 69% lived with other people (Table 2)*.* A sample description can be found in Table 2.

**Table 2:** Socio-demographic and clinical variables of the total sample (*N *= 33).

Socio-demographic and clinical variables	*N* (%)	Mean ± SD
Age		75.2 ± 7.7
65–74	18 (54.5)	
≥75	15 (45.5)	
Gender		
Male	21 (63.6)	
Female	12 (36.4)	
Country		
Italy	21 (63.6)	
Germany	5 (15.2)	
Denmark	7 (21.2)	
Years of schooling		8.7 ± 4.2
Living alone^a^	9 (31.0)	
Weight (kg)		83.1 ± 21.8
Height (cm)		170.4 ± 10.9
Years with cardiac disease		13.6 ± 13.7
NYHA class II symptoms^b^	16 (76.2)	1.95 ± 0.5
LVEF (≤ 40%)^b^^,^^d^	11 (52.4)	
Comorbidities^a^		
Two	13 (44.8)	
≥Three	16 (55.2)	
Medical conditions^c^		
Cardiac arrhythmia		
Yes	19 (57.6)	
No	7 (21.2)	
I do not know	3 (9.1)	
Missing	4 (12.1)	
Old Myocardial Infarction		
Yes	15 (45.4)	
No	14 (42.4)	
I do not know	2 (6.1)	
Missing	2 (6.1)	
High cholesterol		
Yes	12 (36.4)	
No	13 (39.4)	
I do not know	0 (0.0)	
Missing	8 (24.2)	
High blood pressure		
Yes	11 (33.3)	
No	13 (39.4)	
I do not know	0 (0.0)	
Missing	7 (21.2)	
Heart valve disease		
Yes	9 (27.3)	
No	16 (48.5)	
I do not know	4 (12.1)	
Missing	4 (12.1)	
Diabetes mellitus		
Yes	6 (18.2)	
No	19 (57.6)	
I do not know	1 (3.0)	
Missing	7 (21.2)	
Stroke		
Yes	2 (6.1)	
No	27 (81.8)	
I do not know	2 (6.1)	
Missing	2 (6.1)	
Cardiac interventions		
Defibrillator	12 (38.7)	
Bypass surgery	8 (25.8)	
Family history of any heart disease	11 (42.3)	
Hospitalization for a cardiovascular disease	28 (90.3)	

^a^ These data are calculated on a sample of *N *= 29 since data on comorbidities of 4 patients were missing/incomplete.

^b^ These data are calculated on a sample of *N *= 21 since data on NYHA class and LVEF of 12 patients were missing.

^c^ These data refer to the International Classification of Diseases 10th Revision specified with ICD-10 codes (31): Cardiac arrhythmia: ICD-10: I49.9, Old Myocardial Infarction: ICD-10: I25.2, High cholesterol: ICD-10: E78.00, High blood pressure: ICD-10: I10, Heart valve disease: ICD-10: I38, Diabetes mellitus: ICD-10: E14, Stroke: ICD-10: I64.

^d^ Left Ventricular Ejection Fraction ≤40%.

### 3.1 Healthcare needs and preferences in the overall sample

#### 3.1.1 Education

More than a half of the sample (55%) required more information about different aspects related to their diseases (among these, 56% optioned HF); 23% of the total sample reported the need of further information on a healthy lifestyle (33% optioned sleep and 33% physical exercise); 23% about common medical comorbidities (33% optioned diabetes and 28% osteoarthritis). In addition, the most favorite way to get information on their health was through the CM him/herself (79%). Table 3 provides an overview on healthcare needs and preferences of the total sample.

**Table 3:** Healthcare needs and preferences of the total sample (*N *= 33).

Healthcare needs and preferences	*N* (%)
Education	
Area to get more information (*N *= 31)	
Lifestyle	7 (22.6)
Topics around the disease	17 (54.8)
Common condition	7 (22.6)
Most important subject within lifestyle (*N *= 21)	
Exercise	7 (33.3)
Diet	5 (23.8)
Stress	2 (9.5)
Sleep	7 (33.3)
Most important subject within information about topics (*N *= 27)	
Heart failure	15 (55.6)
Emotional distress	3 (11.1)
Having several medical conditions at the same time	9 (33.3)
Most important subject within information about conditions (*N *= 18)	
Hypertension	2 (11.1)
Diabetes type II	6 (33.3)
Osteoarthritis	5 (27.8)
Chronic obstructive pulmonary disease	2 (11.1)
Chronic kidney disease	3 (16.7)
Favourite means of communication	
Text on a website	2 (6.1)
Brochure by medical organizations	5 (15.2)
Video clip	0 (0)
Audio file	0 (0)
Education through your care manager	26 (78.8)
Individual Tailoring of Treatment	
Area of treatment to be more personalized (*N *= 27)	
Medical treatment	5 (18.5)
Personal life	5 (18.5)
Ability to actively engage in improving health	11 (40.7)
Treatment burden	6 (22.2)
Most important subject within medical treatment (*N *= 13)	
Changes in treatment	7 (53.8)
Consideration of alternative treatments	1 (7.7)
More centralized treatment	5 (38.5)
Most important subject within personal life (*N *= 17)	
Quality of life	8 (47.1)
Social life	3 (17.6)
Ability to work	2 (11.8)
Leisure time activities	4 (23.5)
Most important subject within ability to actively engage in improving health (*N *= 17)	
Physical activity	2 (11.8)
Change diet	5 (29.4)
Engage in therapies	5 (29.4)
Self-management	5 (29.4)
Most important subject within treatment burden (*N *= 11)	
Number of appointments	6 (54.5)
Different therapy modules	5 (45.5)
Monitoring	
Aspect of diseases checked by care manager (*N *= 30)	
Symptoms	23 (76.7)
Medical information	2 (6.7)
Medication prescriptions	3 (10.0)
Following treatment recommendations	2 (6.7)
Support	
Area supported by care manager (*N *= 27)	
Adaptation of treatment plan	7 (25.9)
Health behaviours	4 (14.8)
Reduction of emotional burden	5 (18.5)
Communication with GP/informal carer	11 (40.7)
Coordination	
Aspect in need of care manager help (*N *= 27)	
Updates	17 (63.0)
Collaboration with informal carer in the management of health	3 (11.1)
Assistance in finding necessary treatment specialists	3 (11.1)
Assistance in finding community resources	2 (7.4)
Specialist referrals	1 (3.7)
Non-medical	1 (3.7)
Communication	
How to communicate with care manager (*N *= 31)	
Occasional in person meeting [together with general practitioner (GP)]	11 (35.5)
Telephone	18 (58.1)
Video call	2 (6.5)
Other communication paths (*N *= 13)	
Patient dashboard	1 (7.7)
Patient dashboard with a communication function (e.g., emails) to care manager	6 (46.2)
Mobile phone messenger services	6 (46.2)
Online chat forums	0 (0)

#### 3.1.2 Individual tailoring of treatment

Forty-one percent of the patients would like their treatment to be more personalized regarding how to increase their own abilities to engage in their health management and improvement. This includes preferences for changing diet (29%), engaging in prescribed therapies (29%) and autonomous management (29%). A smaller percentage of patients (22%) expressed the need for a re-modulation of their treatment burden, particularly in terms of a higher number of appointments required (55%). The remaining patients expressed the need for a better tailoring of the medical treatment (18.5%), in particular requesting for changes in treatment (54%), or taking into consideration aspects of patient's personal life (18.5%), such as quality of life (47%) (Table 3).

#### 3.1.3 Monitoring

The majority of the sample (77%) would like the CM to systematically monitor patients’ reported symptoms (such as shortness of breath, weight gain, blood pressure, glucose, cholesterol level and other lab results), whereas the remaining patients asked for a monitoring of medication prescriptions (10%), medical information (7%) or adherence to treatment recommendations (7%) (Table 3).

#### 3.1.4 Support

Forty-one percent of the sample asked for support from their CM in communication with their GP and/or informal carer, whereas around a quarter of the patients would like the CM to help them in translating the treatment plan/s (suggested by the physician/s) into their daily routine. Only a minority of the participants expressed the need for support in emotional burden reduction (18.5%) and healthy behaviors adoption (15%) (Table 3).

#### 3.1.5 Coordination

Most of the patients (63%) would like the CM to update their GP about the progression of their symptoms and health behaviors. The rest of the sample expressed the need of a CM who might collaborate with informal carer in patient's health management (11%), assist in finding treatment specialists when needed (11%) or community resources (e.g., self-help groups, cardiac groups) (7%), refer to specialists (4%) and help with non-medical problems (e.g., transportation, payment for medication, financial stress) (4%) (Table 3).

#### 3.1.6 Communication

Fifty-eight percent of the sample preferred telephone contacts with the CM, whereas 36% preferred occasional in-person meetings, involving GP as well. Concerning modern technologies, 46% of the patients would like to use a patient dashboard (e.g., a screen to track/view relevant symptoms and upcoming appointments) with a communication function (e.g., e-mails) to interact with CM; 46% expressed a preference for mobile phone messaging services (Table 3).

### 3.2 Associations between healthcare needs/preferences, sociodemographic variables and number of comorbidities

#### 3.2.1 Sociodemographic variables

No significant differences in healthcare needs according to age were found, whereas a significant difference in relation to preferable communication paths with the CM, according to sex, was found (*χ*^2^ = 6.291, *p *= 0.043). Specifically, 70% of male patients preferred phone calls, whereas 64% of female patients occasional in person meeting, possibly involving GP as well. Table 4 provides healthcare needs and preferences according to gender.

**Table 4:** Healthcare needs and preferences according to gender (*N *= 33).

Healthcare needs and preferences	Gender		*χ* ^2^	*p*
	Male	Female		
	*N* (%)	*N* (%)		
Education				
Area to get more information (*N* = 31)			1.39	0.499
Lifestyle	3 (15.8)	4 (33.3)		
Topics around the disease	11 (57.9)	6 (50.0)		
Common condition	5 (26.3)	2 (16.7)		
Most important subject within information about lifestyle (*N* = 21)			3.73	0.292
Exercise	3 (25.0)	4 (44.4)		
Diet	4 (33.3)	1 (11.1)		
Stress	2 (16.7)	0 (0)		
Sleep	3 (25)	4 (44.4)		
Most important subject within information about topics (*N* = 27)			2.03	0.362
Heart failure	11 (64.7)	4 (40.0)		
Emotional distress	2 (11.8)	1 (10.0)		
Having several medical conditions at the same time	4 (23.5)	5 (50.0)		
Most important subject within information about conditions (*N* = 18)			5.04	0.283
Hypertension	0 (0)	2 (25.0)		
Diabetes type II	5 (50.0)	1 (12.5)		
Osteoarthritis	2 (20.0)	3 (37.5)		
Chronic obstructive pulmonary disease	1 (10.0)	1 (12.5)		
Chronic kidney disease	2 (20.0)	1 (12.5)		
Favourite means of communication (*N* = 33)			2.38	0.304
Text on a website	2 (9.5)	0 (0)		
Brochure by medical organizations	2 (9.5)	3 (25.0)		
Video clip	0 (0)	0 (0)		
Audio file	0 (0)	0 (0)		
Education through your Care Manager	17 (81.0)	9 (75.0)		
Individual tailoring of treatment				
Area of treatment to be more personalized (*N* = 27)			1.08	0.783
Medical treatment	3 (17.6)	2 (20.0)		
Personal life	4 (23.5)	1 (10.0)		
Ability to actively engage in improving health	7 (41.2)	4 (40.0)		
Treatment burden	3 (17.6)	3 (30.0)		
Most important subject within medical treatment (*N* = 13)			4.31	0.116
Changes in treatment	6 (75.0)	1 (20.0)		
Consideration of alternative treatments	0 (0)	1 (20.0)		
More centralized treatment	2 (25)	3 (60.0)		
Most important subject within personal life (*N* = 17)			1.15	0.766
Quality of life	5 (41.7)	3 (60.0)		
Social life	2 (16.7)	1 (20.0)		
Ability to work	2 (16.7)	0 (0)		
Leisure time activities	3 (25.0)	1 (20.0)		
Most important subject within ability to actively engage in improving health (*N* = 17)			2.14	0.544
Physical activity	2 (20.0)	0 (0)		
Change diet	2 (20.0)	3 (42.9)		
Engage in therapies	3 (30.0)	2 (28.6)		
Self-management	3 (30.0)	2 (28.6)		
Most important subject within treatment burden (*N* = 11)			2.21	0.137
Number of appointments	5 (71.4)	1 (25.0)		
Different therapy modules	2 (28.6)	3 (75.0)		
Monitoring				
Aspect of diseases checked by care manager (*N* = 30)			2.51	0.474
Symptoms	15 (78.9)	8 (72.7)		
Medical information	1 (5.3)	1 (9.1)		
Medication prescriptions	1 (5.3)	2 (18.2)		
Following treatment recommendations	2 (10.5)	0 (0)		
Support				
Area supported by care manager (*N* = 27)			0.67	0.879
Adaptation of treatment plan	5 (31.3)	2 (18.2)		
Health behaviours	2 (12.5)	2 (18.2)		
Reduction of emotional burden	3 (18.8)	2 (18.2)		
Communication with GP/informal carer	6 (37.5)	5 (45.5)		
Coordination				
Aspect in need of care manager help (*N* = 27)			7.18	0.207
Updates	10 (62.5)	7 (63.6)		
Collaboration with informal carer in the management of health	3 (18.8)	0 (0)		
Assistance in finding necessary treatment specialists	2 (12.5)	1 (9.1)		
Assistance in finding community resources	0 (0)	2 (18.2)		
Specialist referrals	0 (0)	1 (9.1)		
Non-medical	1 (6.3)	0 (0)		
Communication				
How to communicate with care manager? (*N* = 31)			6.29	0.043*
Occasional in person meeting [together with general practitioner (GP)]	4 (20.0)	7 (63.6)		
Telephone	14 (70.0)	4 (36.4)		
Video call	2 (10.0)	0 (0)		
Other communication paths (*N* = 13)			9.65	0.008*
Patient dashboard	0 (0)	1 (16.7)		
Patient dashboard with a communication function (e.g., emails) to care manager	1 (14.3)	5 (83.3)		
Mobile phone messenger services	6 (85.7)	0 (0)		
Online chat forums	0 (0)	0 (0)		

*p value <0.05.

Also concerning modern technologies, a significant difference according to sex was detected (*χ* ^2^= 9.647, *p *= 0.008). Specifically, 86% of male patients, who addressed this sub-topic, expressed their preference for mobile phone messaging services, whereas 83% of women preferred patient dashboard for communicating with a CM (Table 4).

#### 3.2.2 Number of comorbidities

No significant differences in healthcare needs according to number of comorbidities were found.

## 4 Discussion

This study investigated healthcare needs and preferences of elderly HF patients with multiple comorbidities through a methodology based on the use of cue cards. Patients' needs related to education, individual tailoring of treatment, monitoring, support, coordination, and communication, were investigated and analyzed. Four out of six of these topics referred to the involvement of a hypothetical CM. Our study did not reveal significant differences in healthcare needs according to age and number of comorbidities. In the literature, elderly chronically ill patients and those patients with a greater number of comorbidities (32), including depression (33), reported higher likelihood of healthcare needs. However, according to our review, there is a lack of studies showing significant differences in healthcare needs in relation to age and number of comorbidities among elderly chronically ill patients.

Regarding educational needs, the present study found that just over half of the participants preferred more information about both HF and comorbidities, especially diabetes and osteoarthritis, and about one-third of the patients needed additional information on healthy lifestyle, such as sleep and exercise. The relevant role of information in elderly HF patients is well documented in the literature, and it is associated with disease acceptance and engagement in health improvement (21, 34, 35). Moreover, it is worth noting that from 12% to 24% of the patients did not know/did not answer about their medical comorbidities, supporting existing literature (36) highlighting patients' poor awareness concerning their physical health and medication intake. Consequently, CM should play a significant role in addressing this awareness gap, actively engaging patients in education about their physical health and offering support for improved self-management. Other studies in the literature have introduced a CM for treatment purposes (12, 17, 37). However, the samples included in the cited investigations relied on a wider age range.

As for individual tailoring of treatment, more than 40% of participants expressed a preference for active involvement in their health management through dietary changes, therapy engagement, and self-management. CM, guided by the specifically designed ESCAPE manual and bi-weekly monitored by a multiprofessional Specialist Team (including various specialists such as cardiologists, GPs, psychotherapists, psychiatrists, and pharmacologists), through a close collaboration with patients and their GP/cardiologist, formulates a personalized change in treatment with specific goals derived from the patient's needs and offers continuous proactive assistance to achieve them. Furthermore, more than 20% of the participants mentioned the need to re-modulate treatment burden, specifically about the number of appointments (indeed some patients wished a higher number of visits), which is associated with poor individual treatment tailoring. The remaining participants suggested that the treatment should consider aspects of patients' personal life, such as quality of life, and should be modified, especially with regard to medical therapies. These results are in line with the literature, which also suggests that individual tailoring of treatment is beneficial regarding treatment burden management, ameliorates care adaptation to everyday life and engages patients in improving their health (20–22). In addition, some HF patients not only expressed the need for a change of medical treatment, likely associated with polypharmacy and treatment burden (38, 39), but also the need of improving their quality of life. Both illness and quality of life represent the targets of blended or integrated care. Indeed, in HF patients quality of life has been found to be significantly associated with physical, existential, and psychological well-being (5).

Concerning monitoring, more than three quarters of the participants wished that the CM would check symptoms (such as shortness of breath, weight gain, blood pressure, glucose, and cholesterol) as specified in the cards, whereas the rest of the participants preferred assistance with medication prescription, following treatment recommendations and additional medical information. The results are supported by previous studies (20, 21) and highlight the importance of addressing and monitoring needs among elderly HF patients for clinical stability (34).

Results regarding support suggested that more than 40% of the participants mainly reported the need of support by a CM in communication with their GP and more than one quarter in the adaptation of treatment plan/s suggested by their physician/s into their daily routine. In contrast to previous studies (20, 22), needs for emotional burden reduction and health behaviors support, such as healthy sleeping habits, diet diary and walk regimen, were less pronounced in the participants of our sample. Different patients' age, sample size and methodology used to collect data on healthcare needs, could account for the differences between our results and those found in the literature (20, 22). Specifically, with regards to age, previous studies (20, 22) considered a wider age range, including also patients below 50 years old, whereas in the current study only patients aged 65 years and over were enrolled. Therefore, patients of the present study might present with lower confidence in sharing emotional burden with others (including their carers), requesting health behavior support (31), and engaging in health-related activities, such as physical exercise (40). Moreover, even though also Kyriakou et al. (20) reported the importance of patients' support, in terms of need for communication with the GP and informal carer, they do not suggest the CM as a coordinator of this interaction. Indeed, contrary to previous studies (20, 22), our patients were asked about their healthcare needs and preferences specifically considering the involvement of a hypothetical CM figure addressing them. Finally, even though the current investigation focused on a smaller sample, the involvement of a methodology based on cue colored cards could have captured in a more accurate way the most frequent healthcare needs of elderly HF patients (23, 24).

Regarding patients' needs related to coordination, more than half of the participants preferred the CM to update their GP about the progression of their symptoms and health behaviors, while only a smaller number of participants indicated the need to collaborate with their informal carer in the management of their health and to assist them in finding necessary treatments. In contrast to the literature (21), in our study few participants indicated the need for CM help to go to a specialist, to find community resources and to resolve non-medical problems, such as transportation, payment for medications and financial stress. It could be hypothesized that the participants in the present investigation did not require CM support on social and health care assistance needs because half of them were below 73 years (median age) and all of the participants reported a higher number of years of education as average (8.7 years). This might have resulted in a lower need for external support (e.g., CM), a higher socio-economic status and less financial stress among the patients in our sample, in comparison with Pianese and colleagues’ study (21). In the cited study, indeed, all the patients of the sample were older than 75 years and half of them had elementary school education.

In terms of communication needs, the present study suggests that more than half of the participants preferred communication via telephone to obtain information, monitor disease progression and to have continuous contact with a CM, while almost all the remaining participants preferred occasional in-person meetings. In addition, some patients were also interested in the dashboard with communication function with the CM, and mobile phone messaging services. Furthermore, according to the results regarding communication preferences in association with sex, male patients preferred phone calls, whereas female patients occasional in person meetings. Indeed, according to literature (41, 42), men show a preference for technology-based health related communication, including phone and internet use, over women. CMs should be attentive to this preference when delivering personalized intervention in the framework of BCC care in order to enhance communication with a patient. There is a lack of literature considering HF patients’ needs and preferences that should be addressed by CM in the framework of BCC. Further, despite extensive literature about the need of comprehensive communication between GPs, carers and patients, there is a lack of studies assessing sociodemographic differences in communication preferences among elderly HF patients.

The majority of healthcare needs and preferences of elderly HF patients reported in the present study are in line with those mentioned in the literature (20–22). However, they did not provide specific information about them, whereas our study focused not only on general areas of healthcare needs and preferences, but also investigated specific sub-topics within each area, providing a deeper understanding of them. In our sample, we obtained information about the most frequent patients' needs including education about HF and comorbidities, patients' active engagement in their own treatment, communication support with health professionals and communication with CM preferably by telephone, symptoms monitoring and coordinated regular updates about symptoms progression and health behaviors. In the present study, elderly HF patients with multiple comorbidities mainly reported healthcare needs related to practical aspects of their everyday life, which are in line with existing literature (20). However, needs related to social and health care assistance, as visiting specialists, assistance at home and with finances, were less important to the present sample, in contrast with the study conducted by Pianese et al. (21). In comparison with our study, most of the participants in Pianese et al. (21) investigation were elderly (they had to be at least 75 years old to be enrolled), and they all had to present with a diagnosis of advanced HF, which represents a more severe type of the disease. Finally, the mentioned studies (20–22) did not take into consideration a CM in view of a BCC intervention.

### 4.1 Limitations

Limitations of this study include, first of all, its small sample size. The present study represents the PPI investigation conducted just prior to the ESCAPE RCT (25) and was limited in time. Indeed, as a consequence, the number of the patients in the present study cannot be considered as representative of all elderly multimorbid patients with HF. Furthermore, as the study was based on patients' experience, with data collected through a specifically designed questionnaire, the patients could not provide details on all medications they use. Additionally, we did not compare what the patients reported through the questionnaire with information from their medical records. Also, due to the small sample size, observed associations may be affected by sampling bias, in addition to presence of missing data. However, in the current study, we have reported relevant associations that did not derive from a significant lack of data. Furthermore, given the nature of the present investigation, a power analysis was not executed. The predetermination of the power of the test (e.g., cue cards interview) for our sample was not performed since it was the first time that the cited test was used and, in the literature, other studies on needs did not report power analysis. Finally, the fact that the present methodology was tested for the first time and no re-test was performed, might have had an effect on reliability of the results. However, the new methodology adopted in the present study, based on colored cards with multiple themes regarding patients' needs and preferences, was found to be feasible, easy to use among elderly patients to facilitate communication, and ready to be considered by CMs in the ESCAPE RCT (25).

### 4.2 Conclusion

The findings of the present study provide data to develop a patient-centered BCC based on healthcare needs and preferences in old patients with HF and multimorbidity. These results may pave the ground for personalized treatment in elderly patients suffering from multiple chronic conditions.

### 4.3 Implications for policy and practice

The investigation of patients' needs and preferences, taking into consideration potential role of a CM, highlights the importance of patient participation in the development of more holistic treatment strategies, and further informs the planned ESCAPE BCC how to expand the existing care management elements. Based on the results of the study, further exploration of the potential role of a CM in supporting elderly HF patients with complex healthcare needs is recommended.

## Data Availability

The data that support the findings of this study will be available from the corresponding author, CR, upon reasonable request.
