# Supplementary material for: Healthcare needs in elderly patients with chronic heart failure in view of a personalized blended collaborative care intervention: a cross sectional study
Source: Front Cardiovasc Med. 2024 Mar 13;11:1332356. doi: 10.3389/fcvm.2024.1332356 (PMC10965622; doi:10.3389/fcvm.2024.1332356)
Supplement: Supplementary file 1 [file Table1.docx]

Supplementary Material

**Healthcare Needs in Elderly Patients with Chronic Heart Failure in View of a Personalized Blended Collaborative Care Intervention: a Cross Sectional Study**

**Sara Gostoli** **^†^, Francesco Bernardini^†^, Regina Subach, Petra Engelmann, Tiny Jaarsma, Frida Andréasson, Sanne Rasmussen, Trine Thilsing, Natasja Eilerskov, Barbara Bordoni, Diego Della Riva, Stefano Urbinati, Sebastian Kohlmann, Chiara Rafanelli^*^, On behalf of the ESCAPE Consortium**

*** Correspondence:** Corresponding Author: [chiara.rafanelli@unibo.it](mailto:chiara.rafanelli@unibo.it)

1. **Table S1.** ESCAPE Consortium list

| **No** | **Name** | **Short name** | **Country** | **Principal investigator** | **Authors of the current publication** | **Further members of the ESCAPE consortium** |
| --- | --- | --- | --- | --- | --- | --- |
| 1 | Syddansk Universitet | SDU  Odense | Denmark | • Prof. Susanne S. Pedersen  • Jens Søndergaard | • Trine Thilsing  • Sanne Rasmussen | • Susanne S. Pedersen  • Jens Søndergaard  • Sussi Friis Buhl |
| 2 | Region Sjaelland | ZEALCO - Slagelse | Denmark | • Prof. Søren T. Skou |  | • Peter H. Gæde  • Mette Nyberg • Mette Dideriksen • Lars H. Tang • Prof. Søren T. Skou |
|  |  | ZEALCO - Roskilde |  | • Prof. Niels Eske Bruun |  | • Christian Axel Bang  • Gitte E. Ingwersen  • Prof. Niels Eske Bruun |
| 3 | Universitaetsmedizin Goettingen - Georg-August-Universitaet Goettingen - Stiftung Oeffentlichen Rechts | UMG | Germany | Prof. Christoph Herrmann-Lingen |  | • Cornelia Regner • Miriam Sass • Christine von Arnim  • Mohammed Chebbok  • Michael Koziolek  • Kristina Lang  • Sandrin Plewe • Anja Zinke • Angela Knauf • Ralf Tostmann • Aaron Marshall • Tina Krüger • Christoph Herrmann-Lingen   • Christine Zelenak • Jonas Nagel • Kristina Bersch • Tim Friede  • Hendrika Wiedemann • Thomas Asendorf • Florian Walker  • Birgit Herbeck Belnap |
| 4 | Universitaetsklinikum Hamburg-Eppendorf | UKE | Germany | • Prof. Dr. med. Martin Scherer  • Prof. Bernd Löwe | • Sebastian Kohlmann | • Dagmar Lühmann  • Josefine Schulze  • Agata Menzel  • Prof. Dr. med. Martin Scherer  • Prof. Bernd Löwe |
| 5 | Klinikum der Universitaet zu Koeln | UHC | Germany | Prof. Christian Albus |  | • Christian Albus  • Anna Markser • Lisa Derendorf • Stephanie Stock  • Dusan Simic • Dirk Müller |
| 6 | Universitaet Leipzig | ULEI | Germany | Dr. Rolf Wachter |  | • Liska Hoppe  • Irina Müller-Kozarez  • Rolf Wachter |
| 7 | Royal College of Surgeons in Ireland  Beaumont Hospital | RCSI | Ireland | Prof. Dr. Frank Doyle |  | RCSI:  • Frank Doyle  • Jan Sørensen  Beaumont Hospital:  • Brendan McAdam • David Farrell • Helen Claire Cooney |
| 8 | Lietuvos Sveikatos Mokslu Universitetas | LSMU | Lithuania | Dr. Margarita Beresnevaite |  | • Leonas Valius • Romaldas Maciulaitis  • Egle Rumbinaite  • Margarita Beresnevaite |
| 9 | Semmelweis University | SEG | Hungary | • Dr. Klaudia Vivien Nagy  • Dr. Adrienne Stauder |  | • PhD Piroska Balog • Zsuzsa Bernáth-Lukács  • Dr. Andrea Székely  • Adrienne Stauder • Klaudia Vivien Nagy |
| 10 | Azienda Unità Sanitaria Locale (AUSL) di Bologna | BEL | Italy | Dr. Stefano Urbinati | • Stefano Urbinati  • Diego Della Riva • Barbara Bordoni | • Francesco Guolo |
| 11 | Alma Mater Studiorum - Università di Bologna | UNIBO - University of Bologna | Italy | Prof. Chiara Rafanelli | • Chiara Rafanelli • Sara Gostoli • Regina Subach | • Graziano Gigante |
| 12 | Fraunhofer Gesellschaft zur Foerderung der Angewandten Forschung E.V. | Fraunhofer | Germany | Dr. Carlos A Velasco |  | • Martin Breidenbach • Florim Hamiti • Aynur Guluzade • Naguib Heiba • Yehya Mohamad  • Carlos A Velasco |
| 13 | Eurocarers - Association Europeenne Travaillant avec et pour les Aidants Non-Professionnels | EAC | Belgium | Svetlana Atanasova |  | • Svetlana Atanasova  • Stecy Yghemonos  • Olivier Jacqmain |
| 14 | Bundesarbeitsgemeinschaft Selbsthilfe von Menschen mit Behinderung und Chronischer Erkrankung und ihren Angehorigen E.V. | BAG Selbsthilfe | Germany | Dr. Martin Danner |  | • Franziska Hetzer • Martin Danner • Christina Homma |
| 15 | Linkopings Universitet | LIU | Sweden | Prof. Tiny Jaarsma | Tiny Jaarsma  Frida Andréasson |  |
| 16 | ERINN Innovation | ERINN | Ireland | Rochelle Caruso |  | • Rochelle Caruso  • Jane Maher |
| 17 | Yale University New Haven | YALE | USA |  |  | • Matthew M. Burg |

This table lists all consortium members who have contributed to the presented work.
